# Model-Based Reasoning in Humans Becomes Automatic with Training

**DOI:** 10.1371/journal.pcbi.1004463

**Published:** 2015-09-17

**Authors:** Marcos Economides, Zeb Kurth-Nelson, Annika Lübbert, Marc Guitart-Masip, Raymond J. Dolan

**Affiliations:** 1 Wellcome Trust Centre for Neuroimaging, Institute of Neurology, University College London, London, United Kingdom; 2 Max Planck Centre for Computational Psychiatry and Ageing, University College London, London, United Kingdom; 3 Ageing Research Centre, Karolinska Institute, Stockholm, Sweden; Indiana University, UNITED STATES

## Abstract

Model-based and model-free reinforcement learning (RL) have been suggested as algorithmic realizations of goal-directed and habitual action strategies. Model-based RL is more flexible than model-free but requires sophisticated calculations using a learnt model of the world. This has led model-based RL to be identified with slow, deliberative processing, and model-free RL with fast, automatic processing. In support of this distinction, it has recently been shown that model-based reasoning is impaired by placing subjects under cognitive load—a hallmark of non-automaticity. Here, using the same task, we show that cognitive load does not impair model-based reasoning if subjects receive prior training on the task. This finding is replicated across two studies and a variety of analysis methods. Thus, task familiarity permits use of model-based reasoning in parallel with other cognitive demands. The ability to deploy model-based reasoning in an automatic, parallelizable fashion has widespread theoretical implications, particularly for the learning and execution of complex behaviors. It also suggests a range of important failure modes in psychiatric disorders.

## Author Summary

Automaticity develops with task familiarity. One possible explanation is that automaticity arises when performance of the task becomes habitual, or model-free. Here we asked whether goal-directed, or model-based, reasoning could also become automatic, or resistant to distraction. We used a well-characterized task that differentiates model-based from model-free action. We replicate previous findings that distraction strongly impairs model-based reasoning in task-naive subjects. However, in subjects with prior exposure to the task, distraction does not impair model-based reasoning. This suggests that humans can deploy sophisticated and flexible reasoning more extensively than previously thought.

## Introduction

A wealth of experimental data indicates the brain uses at least two distinct decision making strategies in value-guided choice. One involves prospective reasoning about action-outcome contingencies, while the other retrospectively links rewards to actions [1–3]. The interplay between these two choice strategies has substantial clinical implications. For example, over-reliance on habits could lead to inflexible decision-making in addiction [4] and compulsion [5].

A compelling computational account of these two control mechanisms draws on reinforcement learning (RL) theory [1]. In Daw and colleagues' framework, retrospective learning is accomplished with *model-free* strategies in which rewarded actions tend to be repeated, but the underlying structure of the world that gives rise to these rewards is not learned [6] [7]. Prospective reasoning, on the other hand, relies on a learned model of the world to accurately predict the outcomes of actions, even in the face of changing action-reward contingencies [1,7,8]. This is suggested to render *model-based* reasoning more flexible but at a heightened computational cost [3].

Contemporary theories posit that model-based reasoning engages limited-resource executive functions [9] that involve the dorsolateral prefrontal, ventromedial prefrontal and anterior cingulate cortices [10–15]. This is supported by observations that model-based reasoning is impaired under cognitive load [16] or acute stress [17], and following disruption of dorsolateral prefrontal cortex function via TMS [18], with the degree of impairment interacting with baseline working memory capacity.

However, studies of model-based decision-making often utilize tasks in which the stimuli, contingencies and other task parameters are novel to the subject. This raises the possibility that reliance on limited-resource executive functions is not an intrinsic property of model-based reasoning, but is instead a characteristic of reasoning with an unfamiliar model. In everyday life, tasks become "second-nature" with experience and are subsequently more easily used as building blocks for increasingly complex tasks. It remains untested whether this is entirely due to the formation of efficient habits, or if what is "second-nature" can include sophisticated reasoning with a model of the world.

**Fig 1:**
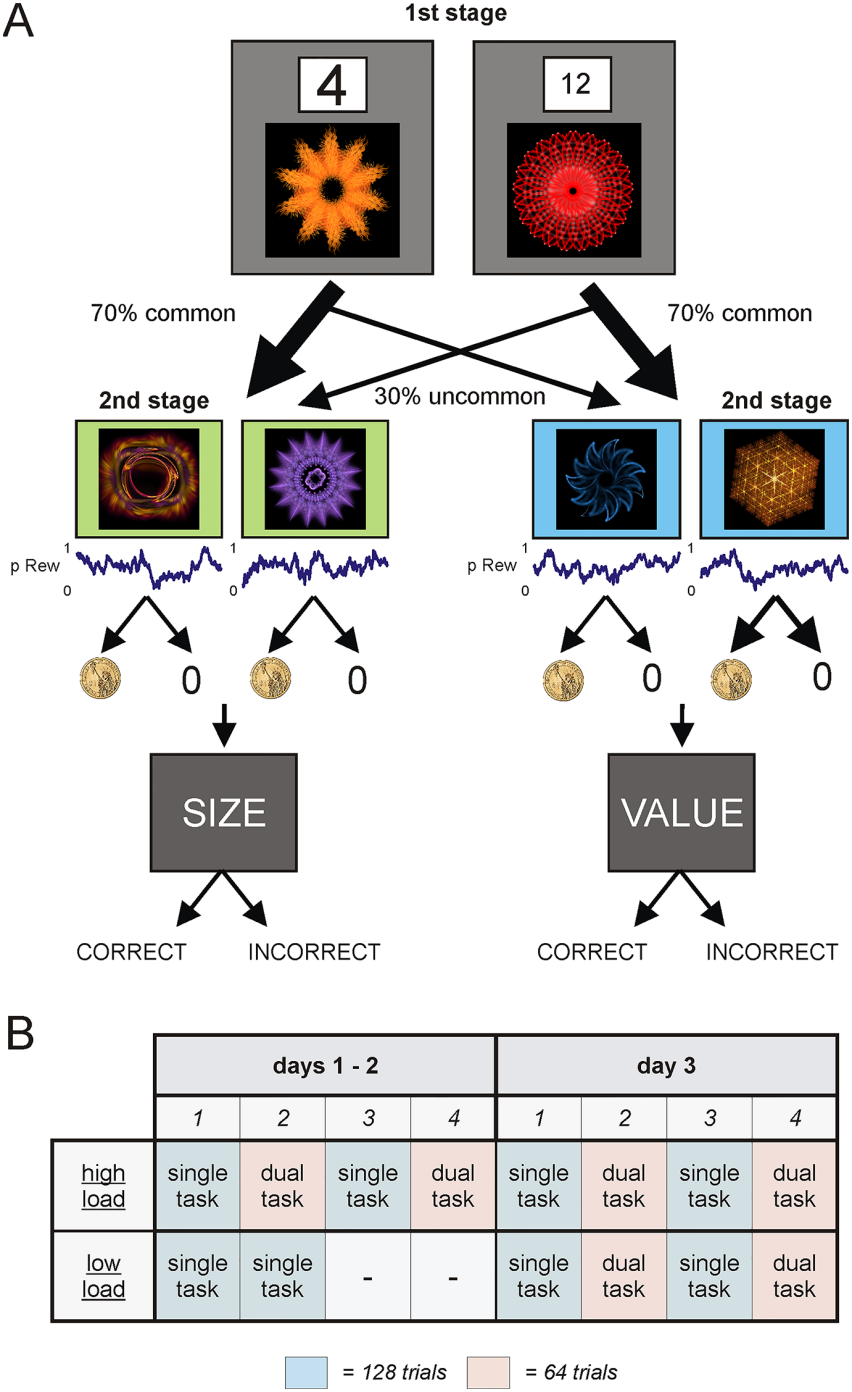
Task and experimental design. (**A**) Subjects chose between a pair of fractals at each of two stages, where a choice at the first-stage lead to one of two second-stage pairs with a fixed probability. This transition structure could be exploited by the player. The second-stage choice followed either a reward (gold coin) or no reward (0), according to independently fluctuating reward contingencies. On dual-task trials (displayed in the figure), two different numbers of physically different sizes were displayed above each fractal at the first-stage. Following second-stage feedback, the word ‘SIZE’ or ‘VALUE’ was presented on the screen, requiring the player to indicate whether the number that was larger in size, or value, respectively, had appeared on the left or right side of the screen. Correct responses were incentivized via monetary gain; incorrect responses were unrewarded. (**B**) On days 1 and 2 the ‘high load group’ played alternating blocks of single-task (128) and dual task (64) trials (for a total of 4 blocks), while the ‘low load group’ played 2 consecutive blocks of single-task (128) trials. On day 3 both groups played alternating blocks of single-task and dual task trials (as per the ‘high load group’ on days 1–2).

Here, we used a two-step decision-task that engages both model-free and model-based reasoning [16,19]. In brief, trials consist of two stages, where each stage involves a two-alternative forced choice between a pair of adjacent fractals (Fig 1). Each first-stage fractal is predominantly associated (with a 70% probability) with one of two second-stage pairs. Transitions with 70% probability we call "common"; those with 30% probability we call "uncommon". The four second-stage fractals are associated with different reward probabilities that fluctuate independently across a session. Thus, subjects have to make trial-by-trial adjustments in choice so as to maximize the probability of reward.

Model-free and model-based decision strategies make different predictions about choice dependence on transitions and rewards from previous trials. We used computational modeling and logistic regression to quantify the contribution of model-free and model-based strategies when subjects performed the two-step task, either alone (single-task condition) or in combination with a demanding concurrent task (dual-task condition). The latter represents a high load condition. We also wanted to test whether the effect of load changed with practice.

To this end we trained subjects on the two-step task for 3 consecutive days and introduced intermittent periods of high load. An initial group of 22 healthy subjects, referred to as the ‘high load group’, experienced the dual-task condition on each day of training. This allowed us to characterize choice under load across the entire training period. A second group of 23 healthy subjects, referred to as the ‘low load group’, experienced the dual-task condition on day 3 only. This allowed us to determine how training on the two-step task alone would impact choice under load.

We hypothesized that model-based calculations would become less reliant on executive resources following training, independent of whether training included or excluded load, leading to a reduction in the detrimental effect of cognitive load on model-based choice.

## Results

### Computational modeling

We analyzed data using previously described reinforcement learning (RL) models [1,19], including a hybrid model and reduced (nested) versions that captured pure model-free and model-based choice. The hybrid model chose according to a combination of model-free and model-based valuations, weighted by the parameter ***w***, such that ***w*** = 0 corresponded to pure model-free and **w** = 1 to pure model-based. Otto and colleagues [16] found that cognitive load shifted ***w*** towards 0. Our central question was whether this shift would be reduced if subjects had prior training on the two-step task. In other words, we asked whether the difference in **w** between single and dual-task trials on day 3 in the ‘low load group’ was smaller than on day 1 in the ‘high load group’ (a between-group comparison). In this comparison, the groups were matched in level of exposure to the Stroop task and the only manipulation was the amount of prior exposure to the two-step task. A secondary question was whether we could track incremental changes in **w** across days (a within-group comparison).

#### Between-group comparison

**Fig 2:**
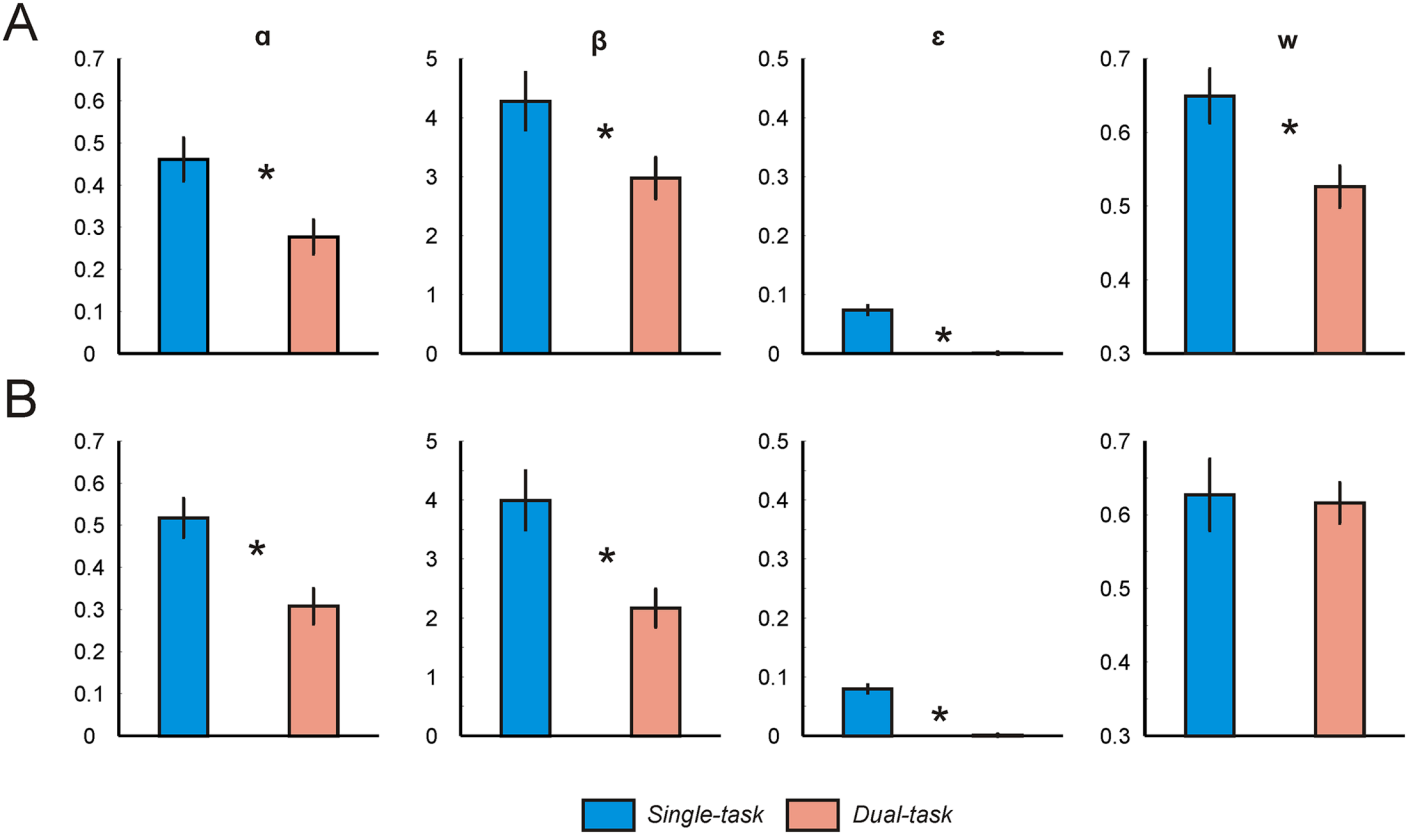
Computational modeling: Between-group comparison. The weighting parameter ***w*** represents a measure of model-based (***w*** = 1) relative to model-free (***w*** = 0) control. ***w*** was lower in the dual-task (high load) condition compared to the single-task (low load) condition in naïve (‘high load group’, day 1) but not trained (‘low load group’, day 3) subjects. Vertical lines represent SEM. * denotes p < 0.05. *α = learning rate*, *β = inverse temperature*, *ε = lapse rate*. (**A**) Mean best-fitting parameters for day 1 of training in the ‘high load group’. (**B**) Mean best-fitting parameters for day 3 of training in the ‘low load group’.

We first sought to validate that choice in the two-step task reflected a mix of both model-free and model-based valuations [19]. We fit the RL models to ‘high load group’ data from day 1 of training, and to ‘low load group’ data from day 3 of training, separately for single-task (two-step alone) and dual-task trials. Using Bayesian model comparison, we found that the hybrid model provided a better fit to subject data in both groups and both trial types, as indicated by a lower iBIC score (see S1 Table). Importantly, in the ‘high load group’ on day 1 the weighting parameter ***w*** was significantly higher in the single-task compared to the dual-task condition (paired t(21) = 2.85, p = 0.01, mean diff = 0.12, 95% CI = [0.03 0.21]), consistent with previous evidence that model-based reasoning is impaired under high cognitive load in untrained subjects [16] (Fig 2A). Conversely, we found no difference in the value of ***w*** between single-task and dual-task trials when fitting ‘low load group’ data from day 3 (paired t(22) = 0.29, p > 0.05) (Fig 2B). This suggests that prior training on the two-step task permitted a strong degree of model-based reasoning under load, despite subjects having no prior experience with performing a task under load.

#### Within-group comparison

**Fig 3:**
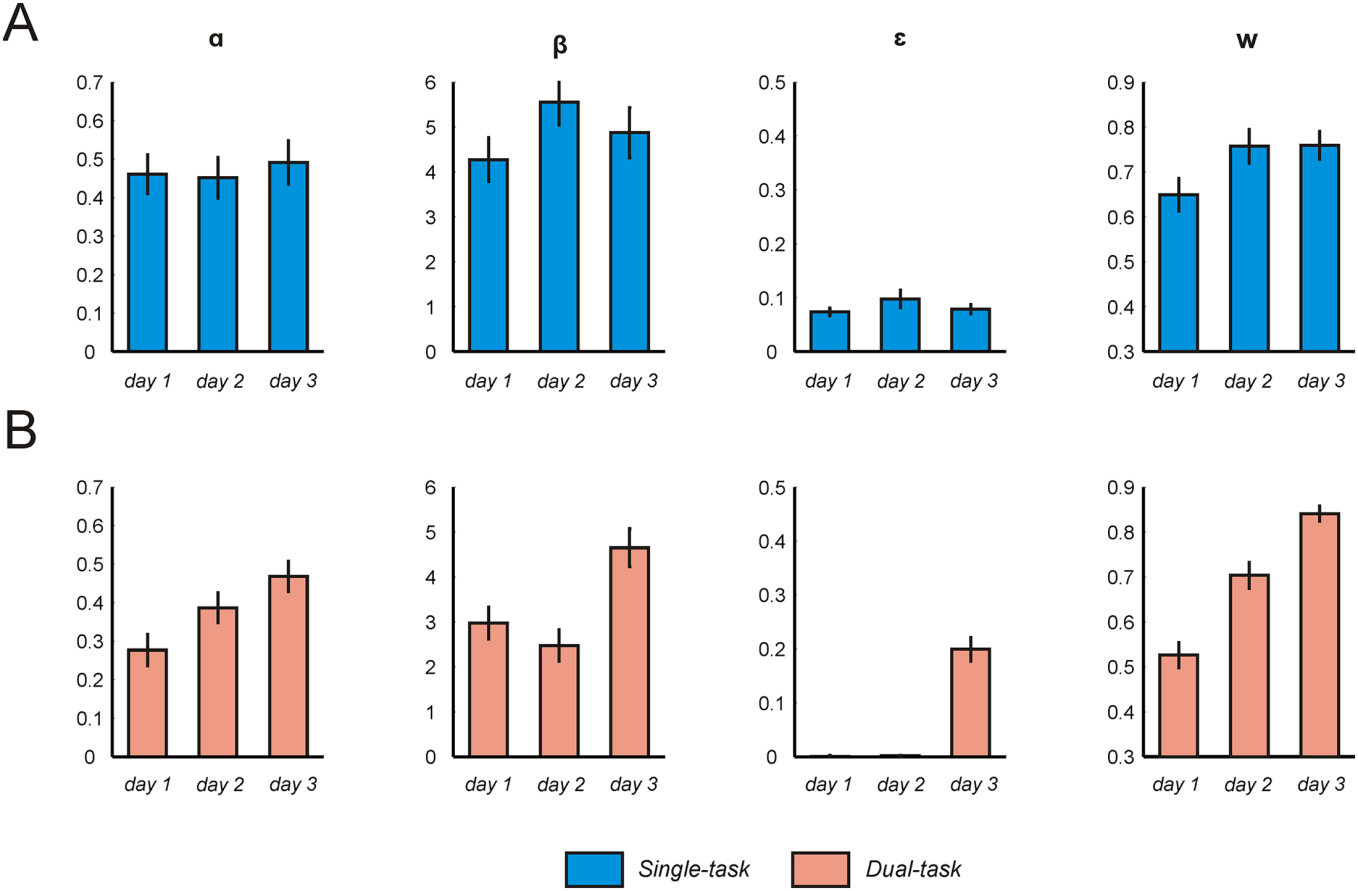
Computational modeling: Within-group comparison. The weighting parameter ***w*** represents a measure of model-based (***w*** = 1) relative to model-free (***w*** = 0) control. At the group level, model parameters remained relatively stable across single-task trials, indicating that performance in the absence of load was modestly influenced by training. By contrast, we observed higher ***w*** values and higher learning rates with increased task exposure during dual-task trials. Vertical lines represent SEM. *α = learning rate*, *β = inverse temperature*, *ε = lapse rate*. (**A**) Mean best-fitting parameters when fitting data from the ‘high load group’ and days 1–3 of training for single-task trials. (**B**) Mean best-fitting parameters when fitting data from the ‘high load group’ and days 1–3 of training for dual-task trials.

Next, we fit the hybrid model to data from days 2 and 3 of training in the ‘high load group’, separately for single-task and dual-task trials. We were interested in whether subjects abruptly switch their choice strategy at the start of a given training day, or alternatively, whether a gradual shift in behavioral control emerges across days. We performed paired t-tests on parameter estimates from Bayesian model inference. In the single-task condition, we found evidence for a moderate shift towards more model-based choice, as indexed by higher ***w*** values on days 2 (paired t(21) = 3.10, p = 0.005, mean diff = 0.11, 95% CI = [0.04 0.18]) and 3 (paired t(21) = 3.66, p = 0.002, mean diff = 0.11, 95% CI = [0.05 0.17]) of training compared to day 1 (Fig 3A). During dual-task trials, we found a more pronounced shift towards model-based choice, with an approximately linear increase in the value of ***w*** across days (Fig 3B). ***w*** was significantly greater on day 2 compared to day 1 (paired t(21) = 4.26, p < 0.001, mean diff = 0.18, 95% CI = [0.09 0.26]), and day 3 compared to day 2 (paired t(21) = 4.08, p < 0.001, mean diff = 0.14, 95% CI = [0.07 0.21]) and 1 (paired t(21) = 9.19, p < 0.001, mean diff = 0.32, 95% CI = [0.24 0.39]). Thus, training increased the relative contribution of model-based reasoning during high load (dual-task) trials, suggesting that the addition of load is necessary to expose training-induced changes in behavior in the two-step task.

#### Multi-day model comparison

To corroborate the finding that ***w*** changes with training within a fully Bayesian framework, we fit a full hybrid RL model (in addition to various nested alternatives) to ‘high load group’ data across all 3 days (combined), separately for single-task and dual-task trials. We tested model variants in which ***w*** could shift across days, governed by a slope parameter ***σ***. Bayesian model comparison revealed an influence of ***σ*** for the dual-task condition but not the single-task condition, with the latter replicating in both cohorts (see S2 and S3 Tables). Thus, training influenced the balance between model-free and model-based control across each day of training in dual-task trials but not in single-task trials (however, we note ***w*** was higher on days 2 and 3 compared to day 1 of training during single-task blocks, a subtlety not captured by a slope model that is only sensitive to linear effects). Importantly, the value of ***σ*** was negative at the group-level, indicating a higher degree of model-based control on day 3 compared to day 1 (see S3 Table). Thus, subjects’ ability to perform model-based reasoning gradually became immune to cognitive load when training included both the single-task and dual-task conditions, both within a fully Bayesian framework, and when fitting behavior from each day individually.

#### Other learning parameters

In addition to differences in the value of ***w*** between single-task and dual-task trials, we found differences in a number of other learning parameters (see Figs 2 and 3, S3 Table). When fitting data from the ‘high load group’ on day 1, and the ‘low load group’ on day 3, we found subjects were less considerate of the most recent reward information (as indexed by a lower learning rate) and chose more stochastically (as indicated by a lower inverse temperature) during dual-task trials compared to single-task trials (high load group ***α***: paired t(21) = 4.33, p < 0.001; high load group ***β***: paired t(21) = 2.94, p = 0.008; low load group ***α***: paired t(22) = 4.61, p < 0.001; low load group ***β***: paired t(22) = 4.49. p < 0.001) (see Fig 2). We identified similar differences when fitting data across all training days consecutively (S3 Table). However, when subjects were able to practice the dual-task condition on each day (‘high load group’), both the learning rate and inverse temperature under load increased across days (***α*** day 2 vs. day 1: paired t(21) = 3.34, p = 0.003; day 3 vs. day 2: paired t(21) = 2.03, p = 0.06; day 3 vs. day 1: paired t(21) = 5.76, p < 0.001; ***β*** day 2 vs. day 1: paired t(21) = -1.45, p > 0.05, day 3 vs. day 2: paired t(21) = 7.96, p < 0.001; day 3 vs. day 1: paired t(21) = 3.84, p < 0.001) (Fig 3B).

### Logistic regression

Computational modeling relies on fitting several model parameters that can exhibit a degree of shared variance, and this has a potential to complicate interpretation when the true value of more than one parameter differs between two conditions. We therefore employed a logistic regression to validate the main findings from our model. We quantified the degree to which choice on the current trial reflected a model-free and model-based influence with respect to events occurring on the preceding 3 trials (see Materials & Methods) [20]. For example, if a player received a reward following an uncommon transition 3 trials in the past, a model-free system would be more likely to repeat the first-stage choice on the current trial, whereas a model-based system would endorse a switch in choice.

**Fig 4:**
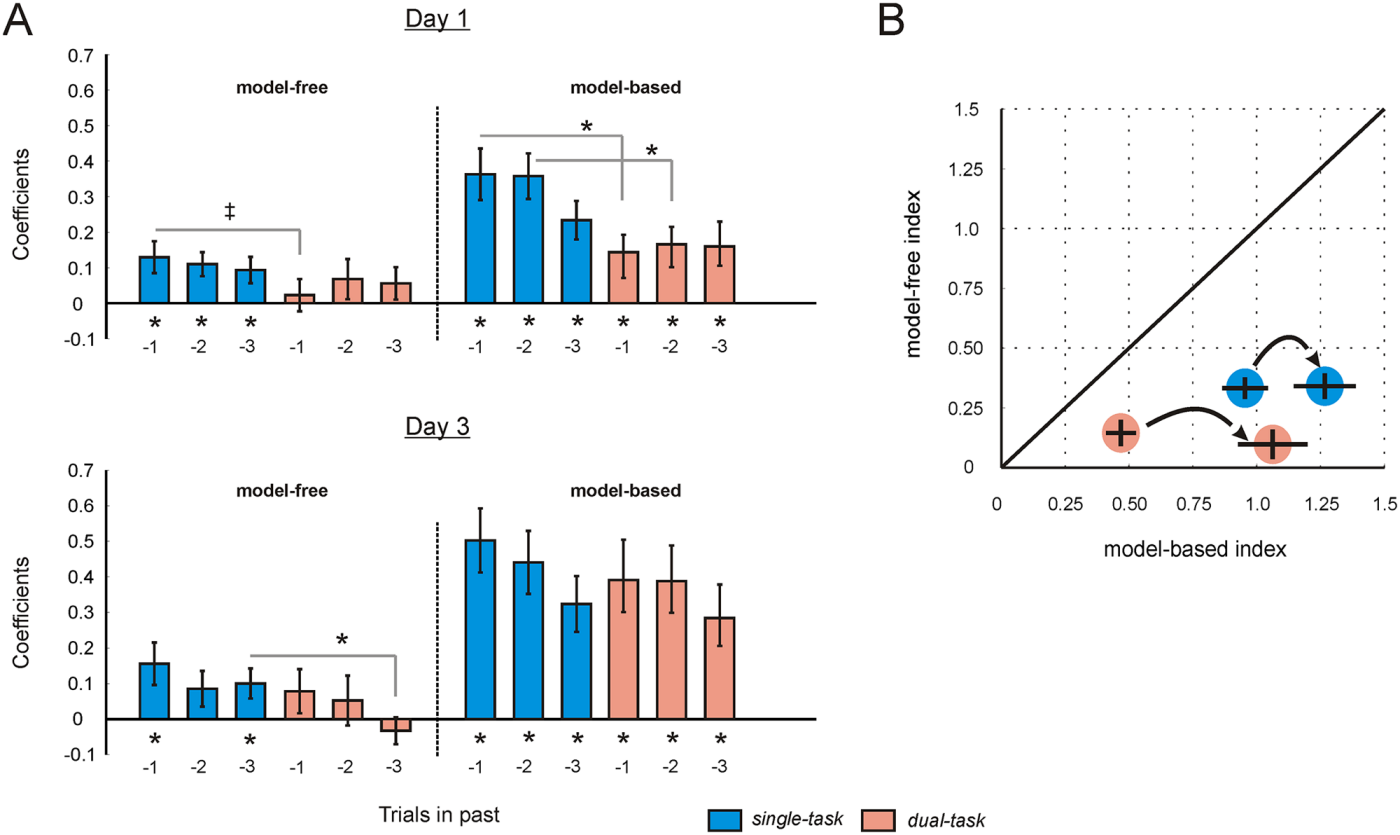
Model-free and model-based influences on choice. Results of a logistic regression that considers model-free and model-based influences on choice in the current trial with respect to events that occurred up to 3 trials in the past. (**A**) Each regressor describes whether events on trial *t*_*-1*_, *t*_*-2*_ and *t*_*-3*_ increase (coded as +1) or decrease (coded as -1) the probability of choosing fractal A according to a model-free or a model-based system (6 total regressors). Model-free coefficients are plotted on the left-hand side of x-axis, and model-based coefficients on the right-hand side. Data from days 1 and day 3 are plotted in the top and bottom panels respectively. Coefficients corresponding to the single-task are shown in blue, and those corresponding to the dual-task are shown in orange. Vertical lines represent SEM. * denotes p < 0.05, ‡ denotes p = 0.09. (**B**) For each condition (single-task in blue, dual-task in orange), and separately for days 1 and 3, we summed (individually) the coefficients corresponding to trial *t*_*-1*_, *t*_*-2*_ and *t*_*-3*_, and derived single estimates of the degree to which model-free (plotted on the y-axis) and model-based (plotted on the x-axis) control were dominant in choice. Vertical lines represent 95% confidence intervals. A line through the origin represents points in which model-free and model-based valuations have an equal influence on choice.

During single-task trials, we identified both a significant model-free and model-based influence on choice extending up to 3 trials in the past (all p < 0.05), consistent with subjects utilizing a hybrid of both systems (Fig 4A). However, we found a reduction in model-based control in the dual-task condition compared to the single-task condition in the ‘high load group’ on day 1, an effect that propagated up to 2 trials in the past (1-back: paired t(21) = 2.59, p = 0.017, mean diff = 0.22, 95% CI = [0.04 0.40]; 2-back: paired t(21) = 2.78, p = 0.011, mean diff = 0.19, 95% CI = [0.05 0.34]). Importantly, this difference was reduced following task training (on day 3), independent of whether training included (‘high load group’, Fig 4A) or excluded (‘low load group’, S1 Fig) the high load condition (high load 1-back: paired t(21) = 1.16, p > 0.05; high load 2-back: paired t(21) = 0.62, p > 0.05). To visualize these effects, we derived single indices of model-free and model-based learning by summing the coefficients that correspond to an influence of events on 1, 2 or 3 trials in the past (see Fig 4B).

To our surprise, we were unable to identify a model-free influence in either group in the high load (dual-task) condition (see Fig 4A and S1 Fig). However, model-free coefficients were not significantly different when comparing the single-task and dual-task conditions (see Fig 4A). Thus, we do not draw strong inferences from this dissimilarity.

In keeping with other studies utilizing the two-step task [16,18,19,21], we repeated the regression analysis but now only considering the influence of events occurring on the immediately preceding trial. Our findings were consistent with the computational modeling approach and the 3-back regression, and are reported in the supplement for completeness (see S2 Fig and S4 Table). In summary, these results replicate our computational modeling in a format with more flexible parametric assumptions.

### Numerical Stroop performance

Mean numerical Stroop accuracy during dual-task trials was 81.9% on day 1, 85.5% on day 2, and 89.5% on day 3 for the ‘high load group’. Thus, performance on the secondary task demonstrated an approximately linear improvement across training days (day 2 vs. day 1: paired t(21) = 2.53, p = 0.019; day 3 vs. day 2: paired t(21) = 3.88, p < 0.001; day 3 vs. day 1: paired t(21) = 5.34, p < 0.001). Mean numerical Stroop accuracy for the ‘low load group’, in which subjects only experienced the dual-task condition on day 3 of training, was 83.2%, and thus comparable to the ‘high load group’.

## Discussion

Here we asked whether reliance on finite executive resources [13,14,18,22–24] is a universal property of model-based reasoning, or whether, as task familiarity increases, model-based reasoning can depend less on these limited-resource functions. We found that reasoning was preserved under load in subjects who had acquired familiarity, through prior training, with the structure of a two-stage Markov decision task [19]. This was replicated in two cohorts of subjects (who received training either with or without load) using different methodological approaches. Our results show that training can enable model-based reasoning even when executive resources are devoted to another task, thereby reflecting the emergence of resource independence.

There are several possible accounts for these findings. First, subjects may change the way they calculate the contingencies of the task following training. From a neural perspective, model calculations may be implemented in new brain areas such that they no longer overlap with those used in the concurrent task. Training has previously been shown to cause "off-loading" in tasks requiring executive resources, including an implementational shift from prefrontal to parietal and striatal regions [25,26]. It is also possible that model calculations remain in the same brain regions, but that coding within these areas becomes more efficient. For example, only a fraction of the initial pool of neurons may be required to realize the same representational fidelity [27–29].

Second, resilience to load could emerge if auxiliary processes (other than reasoning with the structure of the task itself) become more efficient. For example, some cognitive resources may be required for identifying the various stimuli, for tracking events that occurred on previous trials, and for recalling learned values at the second stage. There may also be resource requirements for maintaining belief distributions over meta-parameters, such as whether the task structure changes or new fractals appear, what appropriate learning rates are, when model-based reasoning should be deployed [1,30] and how attentional resources should be allocated within a trial. Since all these depend on executive brain regions to some degree [31–35], a gain in efficiency across any of these domains is likely to free resources.

Third, subjects might learn to perform model-based calculations at the end of each trial ("offline"), rather than at the beginning of the next trial. When used to update a cached or habitual value accessed for the next choice, such offline calculation could relieve the need to store the current reward in memory until the beginning of the next trial. In turn, this might allow better allocation of executive resources to the concurrent task. Indeed, a recent experiment has suggested that the model-based system can “train” the model-free system by replaying and simulating experience offline, and that this in turn allows for choice under load that appears model-based [36].

A final consideration is that choice under load after training may not be truly model-based. Increasingly sophisticated choice heuristics (for example, applying Q-value updates to the opposite first-stage transition following an uncommon transition), can permit behavior that is increasingly difficult to distinguish from fully model-based in the two-step task [37]. Although not realizing the full Markov model of the task, these strategies implicitly embody partial models of task structure. While our data do not adjudicate between these divergent mechanisms, future experiments could aim to investigate their respective predictions using neuroimaging. Further, although our study demonstrates that model-based reasoning can become resist to load, it remains difficult to predict whether these findings would generalize to other task or load manipulations. Indeed, future studies should aim to identify the various factors that might promote or impede such resistance.

Our regression analysis suggests the possibility that the reduction in ***w*** (a parameter indexing the balance between model-based and model-free control) under load could reflect a marginal weakening of model-free reasoning, in addition to a more pronounced disruption of model-based reasoning. This contrasts with previous studies showing that model-based, but not model-free learning, is prone to interference in a range of contexts [5,16–18,38]. This subtle difference may be a consequence of dissimilarities in task design. For example, while Otto and colleagues utilized interleaved trials of low and high load [16], we employed alternating blocks of either condition. If subjects make choices by integrating over the recent trial history, then enforcing a high load over a longer period of trials could have more diffuse consequences on choice.

In addition, we found higher w values on day 3 of training in the ‘high load group’ than the ‘low load group’ in both trial types. Because the 'high load group' had more prior exposure to the Stroop task in this comparison, their higher **w** values could possibly reflect improved facility with the Stroop task itself, or indeed with the performance of any concurrent tasks [3], for example via improved working memory. Thus, we do not draw any strong conclusions from this observation.

In our computational model, load affected not just ***w*** but also prompted slower learning rates and more stochastic choice, independent of training (in the ‘low load group’). The former implies subjects inferred lower environmental volatility under load (perhaps placing stronger weight on priors) [33], or that load induced a tradeoff between working memory and more incremental learning processes that exhibit longer time-constants. More stochastic choice might reflect a reduction in decision confidence [39,40]. It is also possible that the underlying choice strategy used by subjects was not fully captured by our models, leading to some other form of variability to be absorbed by our parameters.

At first glance our result might appear contrary to a standard view that increasing training produces a shift from goal-directed (model-based) to habitual (model-free) control. For example, it is well established that extended training reduces sensitivity to outcome devaluation [13,41–44]. However, our experiment differs from these previous studies as subjects do not receive extended training with a particular action-reward contingency. Instead, they received training with a more sophisticated pattern of relationships between action and reward corresponding to the task structure. This difference appears to be essential for determining whether habits or model-based reasoning are strengthened with experience. Notably, although we conclude that there exist certain conditions where training can improve model reasoning under load, an important remaining question concerns the precise sets of conditions—complexity of model, type of training, and degree of load—whereby this training effect is enhanced or diminished.

A central feature of human learning is the ability to acquire very complex task structures, which often involve performing multiple subtasks in parallel. One way to achieve this parallelism is to reduce the subtasks to habits, reflecting fixed and inflexible action patterns. Our work suggests that even when subtasks are performed in parallel, each subtask can realize sophisticated and flexible model-based reasoning. This lends richness to ideas on the range of behavioral repertoires that humans can express. It is also consistent with the notion of "models" throughout processing hierarchies in the brain, from low-level sensory processing to high-level cognition [45,46].

The possibility that model-based reasoning can become automatic suggests new failure modes (and treatment avenues) in psychiatric disorders. If maladaptive models become automatic, they may lead to behavior that is both sophisticated and pernicious. Conversely, if adaptive models fail to become automatic when they should, they may fail to compete with maladaptive habits, especially under stress or cognitive load. Yet another possible failure mode is that experience calcifies models into true, inflexible habits rather than automatic models.

In summary, we present data that is a challenge to a widespread notion in decision-making that "goal-directed" and "deliberative" are synonymous. We suggest that a dependence of goal-directed reasoning on use of serial executive resources can lessen with task experience. This could be important in the acquisition of progressively more complex behavior, with implications for therapies that aim to restore normal decision-making in psychiatric disorders.

## Materials and Methods

### Ethics statement

Written informed consent was obtained from all participants prior to the experiment and the UCL Research Ethics Committee approved the study (project number 3450/002).

### Subjects

Previous studies in our laboratory and others have shown that 20 to 25 participants provide sufficient power to quantify the contribution of model-free and model-based strategies in the two-step task [16,18,19,38]. We thus decided prior to data collection to include at least 20 participants in the final analysis of each experimental group. 35 adult participants formed a group (referred to as the ‘high load group’) which received training both with and without cognitive load, of which 22 were included in the final analysis (7 male and 15 female; age range 18–34; mean 21.5, SD = 3.71 years). 30 adult participants formed a second independent group (referred to as the ‘low load group’) for which cognitive load was omitted from training on days one and two. 23 were included in the final analysis (9 male and 14 female; age range 18–26; mean 21.2, SD = 3.61 years).

#### Subject inclusion/exclusion criteria

In line with [16] we excluded 11 subjects from the ‘high load group’ and 5 subjects from the ‘low load group’ whose accuracy on the Stroop task during dual-task trials was < 70% on any given day so as to ensure participants were in fact attempting to perform both tasks simultaneously. In addition we excluded 2 participants from the ‘high load group’ and 1 participant from the ‘low load group’ who chose the same first-stage fractal on > 90% of trials (on any given day), irrespective of events on the previous trial. Finally we excluded 1 participant from the ‘low load group’ whose probability of repeating a first-stage action following a common-rewarded transition on the previous trial was < 0.25 on day one of training.

### General design

In the ‘high load group’, subjects performed alternating blocks of single-task (two-step alone) (128 trials) and dual-task (64 trials) trials until two blocks of each trial type were completed (256 single-task trials, 128 dual-task trials in total). This protocol was repeated across three consecutive days. Subjects received 20 practice trials of each trial type at the start of day one. In the ‘low load group’, subjects performed 256 trials of the single-task (two-step alone) condition for two consecutive days, while the protocol on day three was identical to the ‘high load group’. Subjects in the ‘low load group’ received 20 practice trials of the single-task condition at the start of day one, and 20 practice trials of the dual-task condition at the start of day 3.

### Task

Subjects performed a two-step decision task based on [19] and equivalent to that used in [16]. At the first stage, subjects had 2000 ms to choose between a fractal-pair presented on a grey background (the chosen fractal was highlighted with a yellow border for the remainder of the choice period). Each first stage fractal led to one of two second stage fractal-pairs with a 70% probability (common transition) and to the other with a 30% probability (uncommon transition). Second stage fractal-pairs were displayed on a green or blue background in accordance with whether a common or uncommon transition had occurred. In addition, the chosen first-stage fractal was minimized and moved to the top central portion of the screen. At the second stage, subjects again had 2000 ms to choose between a fractal-pair (the chosen fractal was again highlighted with a yellow border for the remainder of the choice period). An outcome was presented in the form of a golden coin (to indicate a monetary gain) or a ‘0’ (to indicate no monetary gain), followed by an inter-trial interval (fixation cross). The position of each fractal (left versus right) was counter-balanced across trials for both stages.

Dual-task trials followed the same procedure, except that subjects had to simultaneously perform a numerical Stroop task [47]. At the beginning of the first stage, two digits were presented, one above each choice fractal, for 200 ms, and then covered by a white mask for a further 200 ms. After second-stage choice feedback, either the word ‘SIZE’ or ‘VALUE’ appeared alone in the center of the screen on a grey background. The player had 1000 ms to indicate which first-stage number was larger in size or value respectively. In accordance with [16] and [47], the numerically larger number was physically smaller on 85% of trials. Thus, subjects had to hold incidental information in working memory whilst performing the two-step task. Following their response, feedback in the form of the word ‘CORRECT’ or ‘INCORRECT’ was presented a further 1000 ms. If participants failed to respond during the Stroop task probe, a red “X” appeared for 1000 ms. Trial lengths were equated across two-step and dual task trials (7200 ms per trial).

The reward probabilities associated with second-stage fractals were governed by independently drifting Gaussian random walks (SD = 0.025). We generated a pool of fifteen random walks for which reward probabilities did not exceed ~0.75 or fall below ~0.25. For each subject, three walks were selected at random from the pool for use on each successive day of training. Thus, walks were continuous between blocks of single-task and dual task trials.

### Computational modeling

Based on [19], the task was modelled as consisting of three states (*s*_*A*_ for the first-stage fractal pair; *s*_*B*_ and *s*_*C*_ for the second-stage fractal pairs) where two possible actions (*a*_*A*_,*a*_*B*_) can be taken from each state. The goal of each RL algorithm is to learn a state-action value function *Q*(*s*,*a*) that maps each state-action pair to its expected future value. In each trial *t*, the first and second-stage states are indicated as *s*_1,*t*_ and *s*_2,*t*_ respectively, while first and second-stage choices (actions) are indicated as *a*_1,*t*_ and *a*_2,*t*_ Since there is no reward at the first stage, *r*_1,*t*_ is always zero, while *r*_1,*t*_ can be zero or one.

#### Model-free

The model-free algorithm was temporal difference Q-learning [6] in which the value of a given state is assumed to be equivalent to the expected reward from taking the best available action from that state. At each stage *i* of each trial *t*, the value of the chosen state-action pair was updated according to:

$$Q_{TD}\left(s_{i,t},a_{i,t}\right)=Q_{TD}\left(s_{i,t},a_{i,t}\right)+\;\alpha \delta_{i,t}$$

where *δ*, the reward prediction error (RPE), is defined as

$$\delta_{i,t}=r_{i,t}+\gamma \;\underset{a}{\text{max}}\left[Q_{TD}\left(s_{i+1,t},a\right)\right]-\;Q_{TD}\left(s_{i,t},a_{i,t}\right)$$

where *α* is a learning rate fit for each subject and *γ* is a discount factor that trades off the importance of sooner versus later rewards (fixed at 1).

Note that for the first stage choice, *r*_*i*,*t*_ is always zero and *δ* is instead driven by the second-stage value.

After outcome delivery, the second stage RPE is used to update the first-stage action *Q*_*TD*_(*s*_1,*t*_,*a*_1,*t*_) according to the eligibility trace λ, which assigns credit to the first-stage action without the need for an additional step.

$$Q_{TD}\left(s_{1,t},a_{1,t}\right)=Q_{TD}\left(s_{1,t},a_{1,t}\right)+\;\alpha \lambda \delta_{2,t}$$

Thus, in the event that λ = 0, choice is driven by the estimated value of the second-stage state on the previous trial. Consistent with previous studies [16,19], this model assumes that eligibility traces are cleared between trials.

#### Model-based

A model-based RL algorithm involves learning a set of contingencies between actions and states (a state-transition function), estimating a reward value for each state, and then combining the two by iterative expectation. Here, since first-stage transitions are probabilistic, a player must map action-state pairs to a probability distribution over subsequent states.

One can approximate subjects’ estimate of the transition probabilities by assuming they believe one of two alternatives:

$$P\text{(}s_{B}|\;s_{A}\;,a_{A})=0.7,\;P\text{(}s_{C}|\;s_{A}\;,a_{A})=0.3,\;P\text{(}s_{C}|\;s_{A}\;,a_{B})=0.7,\;P\text{(}s_{B}|\;s_{A}\;,a_{B})=0.3$$

or

$$P\text{(}s_{B}|\;s_{A}\;,a_{A})=0.3,\;P\text{(}s_{C}|\;s_{A}\;,a_{A})=0.7,\;P\text{(}s_{C}|\;s_{A}\;,a_{B})=0.3,\;P\text{(}s_{B}|\;s_{A}\;,a_{B})=0.7$$

based on the number of previous transitions from *s*_*A*_ to *s*_*B*_ given *a*_*A*_ and from *s*_*A*_ to *s*_*C*_ given *a*_*B*_ (or vice versa). A previous study has shown this scheme settles on the true transition matrix after the first few trials and fits subjects’ choices better than implementing a traditional trial-by-trial learning algorithm [19]. Therefore, we assume the true transition probabilities are learnt during practice trials and are known by the start of the first experimental block.

Since the second-stage action is the only choice associated with immediate reward, and is the final step in a trial, an agent can learn the value of the second-stage state in a manner equivalent to temporal difference Q-learning (as above). Thus, *Q*_*TD*_(*s*_2,*t*_,*a*_2,*t*_) is simply an estimate of the immediate reward *r*_2,*t*,_ and the model-based algorithm converges with model-free learning at this stage.

By combining the transition function with the second-stage values we can define the values of the two first-level actions (using Bellman’s equation) as follows:

$$Q_{MB}\left(s_{A},a_{j}\right)=\;P\left(s_{B}|s_{A},\;a_{j}\right)\underset{a}{\text{max}}\left[Q_{TD}\left(s_{B},a\right)\right]+\;P\left(s_{C}|s_{A},\;a_{j}\right)\underset{a}{\text{max}}\left[Q_{TD}\left(s_{C},a\right)\right]$$

where these are computed on every trial based on the updated second-stage Q-values.

#### Hybrid model

For the hybrid model we consider contributions from both model-free and model-based RL. First-stage action values were defined as the weighted sum of values from the algorithms described above as follows:

$$Q_{HM}\left(s_{A},a_{j}\right)=wQ_{MB}\left(s_{A},a_{j}\right)+\left(1-w\right)Q_{TD}\left(s_{A},a_{j}\right)$$

where ***w*** is a weighting parameter.

When fitting data across all sessions, we included a slope parameter sigma (***σ***) that allowed ***w*** to shift across days:

$$w_{D}=w\left[\text{exp}\left(\sigma \left(Day-2\right)\right)\right]$$

and used ***w***_***D***_ as the new weighting parameter.

At the second-stage, all three models (model-free, model-based, hybrid) converge.

#### Action selection

For each model, values were converted to action probabilities using a sigmoid (softmax) function:

$$P\left(a_{A,t}\right)=\varepsilon +\;\left(1-2\varepsilon \right)\frac{\text{exp}\left(\beta \cdot Q\left(s_{i,t},a_{A,t}\right)\right)}{\text{exp}\left(\beta \cdot Q\left(s_{i,t},a_{A,t}\right)\right)+\;\text{exp}\left(\beta \cdot Q\left(s_{i,t},a_{B,t}\right)\right)}$$

Where ***ε*** is a lapse rate, and ***β*** is an inverse temperature parameter that governs the stochasticity of choice options. When ***ε*** > 0 the boundaries of the sigmoid function are compressed and deviations from the model are less harshly punished (see S3A Fig). Including a lapse rate in the softmax may reduce the impact of choices unrelated to the value function of our model (for example choices that result from lapses in concentration, or pressing the wrong button) on the estimation of the remaining parameters (see S1 Text and S3B Fig for further explanation).

#### Model sets

When fitting data from individual days, we considered a hybrid RL model that included a single learning rate (***α***) and softmax temperature (***β***), a weighting parameter that governs the balance between model-free/model-based control (***w***), and a lapse rate (***ε***). The eligibility trace (***λ***) was fixed at 1. Model-free and model-based algorithms were nested versions of the hybrid model where ***w*** was set to 0 and 1 respectively.

When fitting data across all days, we considered a family of (nested) hybrid RL models in which specific parameters were omitted or included as fixed versus free parameters. More complex models included separate RL parameters for first and second stage choices, an eligibility trace, and a slope parameter that permitted the weighting between model-free and model-based control to shift across days. See S2 Table for the full model set.

#### Model fitting and comparison

The model fitting routine follows that previously described by Huys and colleagues [48]. Each model yielded a parameter vector, *θ*_*i*_, for each subject, *i*. Before inference, all parameters were suitably transformed to enforce constraints (log and inverse sigmoid transforms). Model fitting at the individual level aimed to find the maximum a posteriori estimate of *θ*_*i*_, given a vector of each subject’s choices,*C*_*i*_:

$$\theta_{i}=argmax_{\theta }\;p(C_{i}\left|\theta_{i})p(\theta_{i}\right|\vartheta )$$

We used a hierarchical (random effects) model-fitting approach, with the assumption that parameter estimates were normally distributed at the group level, where *ϑ* are the parameters of the empirical normal prior distribution (hyperparameters) on *θ*. The hierarchical approach allows the population-level distribution of data to constrain unreliable parameter estimates at the individual level. We estimated the maximum-likelihood hyperparameters, given the data from all *N* subjects:

$${\hat{\vartheta }}^{ML}=\;argmax_{\vartheta }\;p\left(C_{1}\ldots \;C_{N}|\vartheta \right)=\;argmax_{\vartheta }\underset{i}{\prod^{}}p(C_{i}|\vartheta )$$

where:

$$p\left(C_{i}|\vartheta \right)=\;{\int^{}}^{}d\theta_{i}\;p\left(C_{i}|\theta_{i}\right)p(\theta_{i}|\vartheta )$$

The intractable integral above was estimated by Expectation-Maximization (EM). The E-step at the *k*th iteration sought the maximum a posteriori parameter estimates for each subject (given an estimate of the empirical prior from the preceding iteration, achieved by unconstrained nonlinear optimization in Matlab, Mathworks, MA, USA):

$$\theta_{i}{}^{\left(k\right)}=\;argmax_{\theta }\;p(C_{i}\left|\theta_{i})p(\theta_{i}\right|\vartheta^{\left(k-1\right)})$$

We used a Laplace approximation, which assumes that the likelihood surface is normally distributed around the maximum a posteriori parameter estimate:

$$p\left(\theta_{i}|C_{i}\right)\approx N\left(\theta_{i}{}^{\left(k\right)},{\sum^{}}^{}{}_{i}^{\left(k\right)}\right)$$

Where ${\sum^{}}^{}{}_{i}^{\left(k\right)}$ is the second moment around $\theta_{i}{}^{\left(k\right)}$, which approximates the variance. In the M-step, the estimated hyperparameters *ϑ* ^*(k)*^ of the normal prior distribution, mean *μ*, and factorized variance, *σ* ^2^, were updated as follows:

$$\mu^{\left(k\right)}=\;\frac{1}{N}\underset{i}{\sum^{}}\theta_{i}{}^{\left(k\right)}$$

$${\left(\sigma^{\left(k\right)}\right)}^{2}=\frac{1}{N}\underset{i}{\sum^{}}\left[{\left(\theta_{i}{}^{\left(k\right)}\right)}^{2}+{\sum^{}}^{}{}_{i}^{\left(k\right)}\right]-{\left(\mu^{\left(k\right)}\right)}^{2}$$

We compared models by Bayesian model evidence, *p*(*C*_1_ … *C*_*N*_|*M*), approximated as *BIC*_*int*_:

$$-\frac{1}{2}BIC_{int}=\text{log}p(C_{1}\ldots \;C_{N}|{\hat{\vartheta }}^{ML}\text{)}-\;\frac{1}{2}\left|M\right|\text{log}\left(\left|C_{1}\ldots \;C_{N}\right|\right)$$

Where |*C*_*1*_ … *C*_*N*_| is the total number of choices made by all subjects, and |*M*| is number of hyperparameters fitted. Notably here, by distinction from conventional BIC, $\text{log}p(C_{1}\ldots \;C_{N}|{\hat{\vartheta }}^{ML}\text{)}$ is a sum over the model evidence at the subject level by integrating over subject-level parameters:

$$\text{log}p(C_{1}\ldots \;C_{N}|{\hat{\vartheta }}^{ML}\text{)}=\;\underset{i}{\sum^{}}\text{log}{\int^{}}^{}d\theta \;p\left(C_{i}|\theta \right)\;p\left(\theta |{\hat{\vartheta }}^{ML}\right)\;\approx \;\underset{i}{\sum^{}}\text{log}\frac{1}{K}\overset{K}{\underset{k=1}{\sum^{}}}p\left(C_{i}|\theta^{k}\right)$$

The right hand expression approximates the integral by summing over *K* samples, drawn from the empirical prior, $p\left(\theta |{\hat{\vartheta }}^{ML}\right)$. Thus the individual-level parameters intervene between the data and the group-level inference, but are averaged out when comparing models.

### 3-back logistic regression

In line with recent studies using the two-step task, we considered model-free and model-based influences on choice in the current trial, with respect to events that occurred up to 3 trials in the past [20]. Here, the dependent variable on trial *t* was 1 when stimulus A was chosen and 0 when stimulus B was chosen at the first-stage. Each regressor then described whether events on trial *t*_*-1*_, *t*_*-2*_ and *t*_*-3*_ would increase (coded as +1) or decrease (coded as -1) the probability of choosing A according to a model-free or a model-based system (6 regressors in total). Importantly, if a trial involved a common transition, both systems make identical predictions. However, opposing predictions emerge following uncommon transitions. We implemented a random-effects logistic regression in Matlab (MathWorks) and performed one-sample t-tests on the resulting coefficient estimates for the 6 regressors, separately for trained (day 3) versus untrained (day 1), and dual-task (high load) versus single-task (low load) conditions (see Fig 4 and S2 Fig).

## Supporting Information

S1 Fig Model-free and model-based influences on choice: ‘Low load group’. We performed a logistic regression on data from the ‘low load group ‘ on day 3 of training to estimate the relationship between choice on trial *t* and events occurring on trial *t*_*-1*_ up to *t*_*-3*_. Here, regression coefficients can be interpreted as reflecting a model-free or model-based influence on choice, where larger coefficients indicate a stronger influence. In the single-task condition (blue bars), model-free and model-based coefficients were significantly different from 0 (up to 3 trials in the past), suggesting that subjects used a hybrid of both strategies. In the dual-task (high load) condition (orange bars), we observed a significant influence of a model-based system, that did not differ from the single-task condition, up to 3 trials in the past. In contrast, we found no significant influence of a model-free system. These results are consistent with data from the ‘high load group’ (see Fig 4). Vertical lines represent SEM. * denotes p = < 0.05, ‡ denotes p = 0.08.
(TIF)

S2 Fig Switch-stay choice pairs. Bar plots show the average probability with which subjects chose to repeat their first-stage action on the subsequent trial as a function of the transition (common vs. uncommon) and outcome (rewarded vs. unrewarded) on the previous trial. Blue bars correspond to common transitions and red bars correspond to uncommon transitions. Vertical lines represent SEM. (**A**) Data from the ‘high load group’. The upper panel corresponds to the single-task condition and the lower panel to the dual-task condition. Choice is plotted separately for all 3 days. (**B**) Data from the ‘low load group’. Behavior is plotted across all 3 days for the single-task condition, and for day 3 alone in the dual-task condition.
(TIF)

S3 Fig The effect of utilizing a softmax lapse rate. **(A)** The left-hand side shows an empirical softmax function generated using data from the ‘high load group’ on day 1 and the single-task condition. For each subject, we grouped the values generated from the winning hybrid model (see S1 Table) into 10 bins, and calculated the mean probability with which the best action was chosen in each bin, including both first and second-stage choices. The plot is averaged over all 22 subjects in the ‘high load group’. Vertical bars represent SEM. The right-hand side shows a simulated softmax function with an inverse temperature (***β***) of 1, with and without including a lapse rate (***ε***) set to 0.1. The lapse rate compresses the boundaries of the softmax such that the probability of choosing a given action is forced to lie between the range of 1-2*ε*. **(B)** Here we show slices through the likelihood surface of a single subject when the lapse rate (***ε***) is set to 0 (left-hand side), or fit as a free parameter (right-hand side), respectively. The red crosses represent the peak of the likelihood surface. On the right-hand side, the black arrow represents the shift in the peak of the surface (and the equivalent shift in the best-fitting values of our model parameters) when ***ε*** is fit as a free parameter compared to when it is fixed at 0.
(TIF)

S1 Table Bayesian model comparison: Single days. Results of a Bayesian model comparison that accounted for differences in model complexity. The hybrid model, which incorporated influences from both model-free and model-based control, fit subject data better than pure model-free and model-based RL algorithms across both trial types (single-task versus dual-task) and both groups (‘high load group’ day 1, ‘low load group’ day 3). Bold-face denotes the winning model (lowest iBIC score) for each condition. α = learning rate; β = softmax inverse temperature; ε = lapse rate; w = model-free/model-based weight. The eligibility trace, λ (not shown), was set to 1 in all cases. w was set to 0 and 1 for pure model-free and pure model-based RL respectively.
(DOCX)

S2 Table Bayesian model comparison: Multiple days. Results of a Bayesian model comparison that accounts for differences in model complexity. More complex model variants include those that have separate parameters for first and second stage choices, an eligibility trace, and a parameter for capturing shifts in model-free versus model-based control across days (σ). In simpler models, RL parameters were fixed between first and second stage choices, the eligibility trace was fixed at 1, and σ was set to 0. Bold-face denotes the winning model (lowest iBIC score) for each condition. Parameters followed by a superscript of 1 or 2 correspond to first-stage or second-stage choices respectively. α = learning rate; β = softmax inverse temperature; ε = lapse rate; w = model-free/model-based weight; λ = eligibility trace; σ = slope governing a shift in model-free/model-based weight (w) across days.
(DOCX)

S3 Table Inferred group-level parameters. Best-fitting parameter estimates shown separately for each group and condition (single-task versus dual-task), using data concatenated across all 3 days of training. Values represent mean parameter fits across all subjects. * represents fixed parameter values. Parameters followed by a superscript of 1 or 2 correspond to first-stage or second-stage choices respectively. In simpler models, λ was fixed at 1 and σ was set to 0. α = learning rate; β = softmax inverse temperature; ε = lapse rate; w = model-free/model-based weight; λ = eligibility trace; σ = slope governing a shift in model-free/model-based weight (w) across days.
(DOCX)

S4 Table Results of a logistic regression across days. Table shows the group-level output of a logistic regression on first-stage switch-stay behavior, separately for single-task (‘high load group’ and ‘low load group’) and dual-task trials, from data concatenated across all 3 training sessions. We note that ‘reward x day’ was orthogonalized with respect to reward, and in turn ‘reward x transition x day’ was orthogonalized with respect to ‘reward x transition’. These regressors thus account for variance unexplained by the simpler main effect or 2-way interaction respectively (see Materials & Methods). Bold-face denotes p < 0.05 uncorrected for multiple comparisons. *rew = reward*; *trans = transition*.
(DOCX)

S1 Text Supporting Information. (DOCX)
