# Supplementary figures and images for: Model-Based Reasoning in Humans Becomes Automatic with Training
Source: PLoS Comput Biol. 2015 Sep 17;11(9):e1004463. doi: 10.1371/journal.pcbi.1004463 (PMC4588166; doi:10.1371/journal.pcbi.1004463)

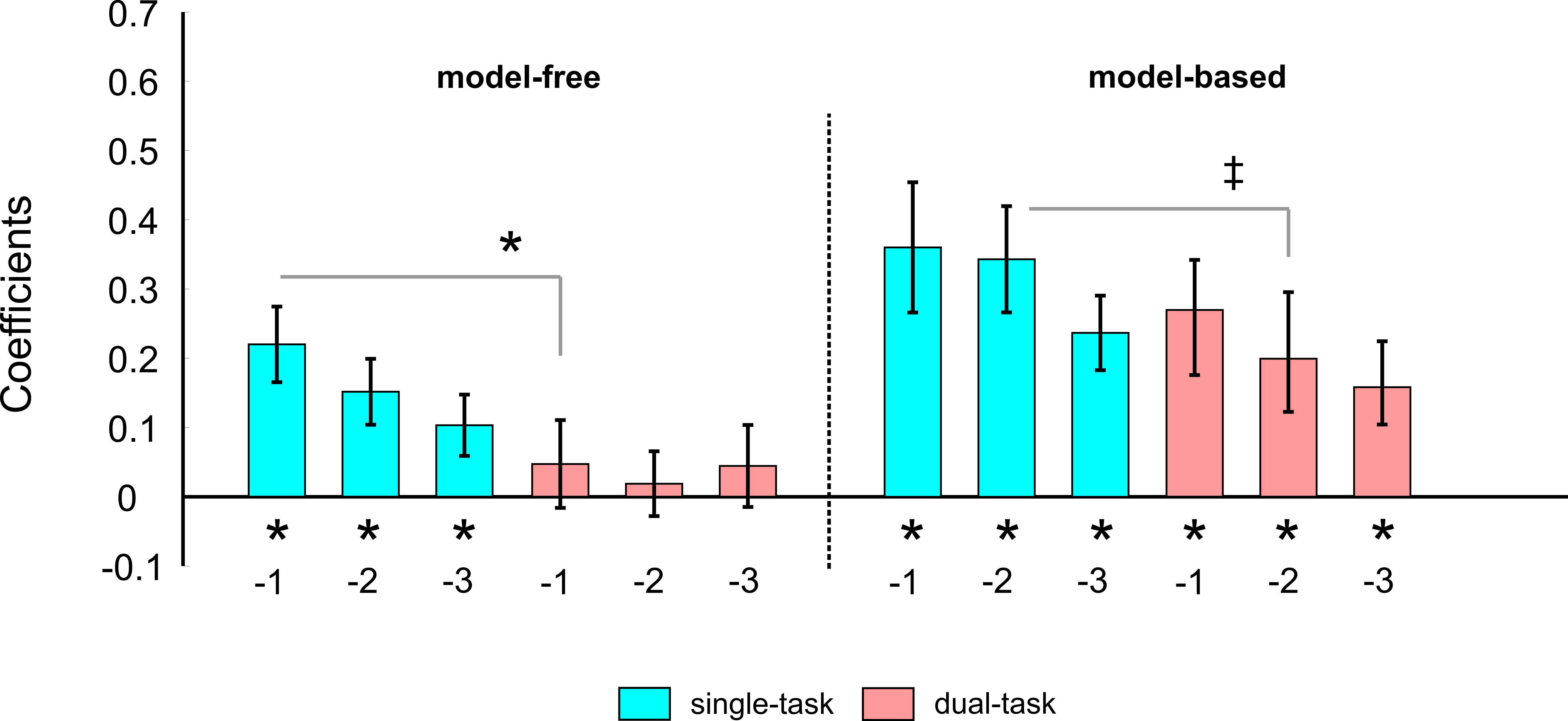

Supplement: S1 Fig — We performed a logistic regression on data from the ‘low load group ‘ on day 3 of training to estimate the relationship between choice on trial t and events occurring on trial t -1 up to t -3. Here, regression coefficients can be interpreted as reflecting a model-free or model-based influence on choice, where larger coefficients indicate a stronger influence. In the single-task condition (blue bars), model-free and model-based coefficients were significantly different from 0 (up to 3 trials in the past), suggesting that subjects used a hybrid of both strategies. In the dual-task (high load) condition (orange bars), we observed a significant influence of a model-based system, that did not differ from the single-task condition, up to 3 trials in the past. In contrast, we found no significant influence of a model-free system. These results are consistent with data from the ‘high load group’ (see Fig 4). Vertical lines represent SEM. * denotes p = < 0.05, ‡ denotes p = 0.08. (TIF) [file pcbi.1004463.s001.tif]

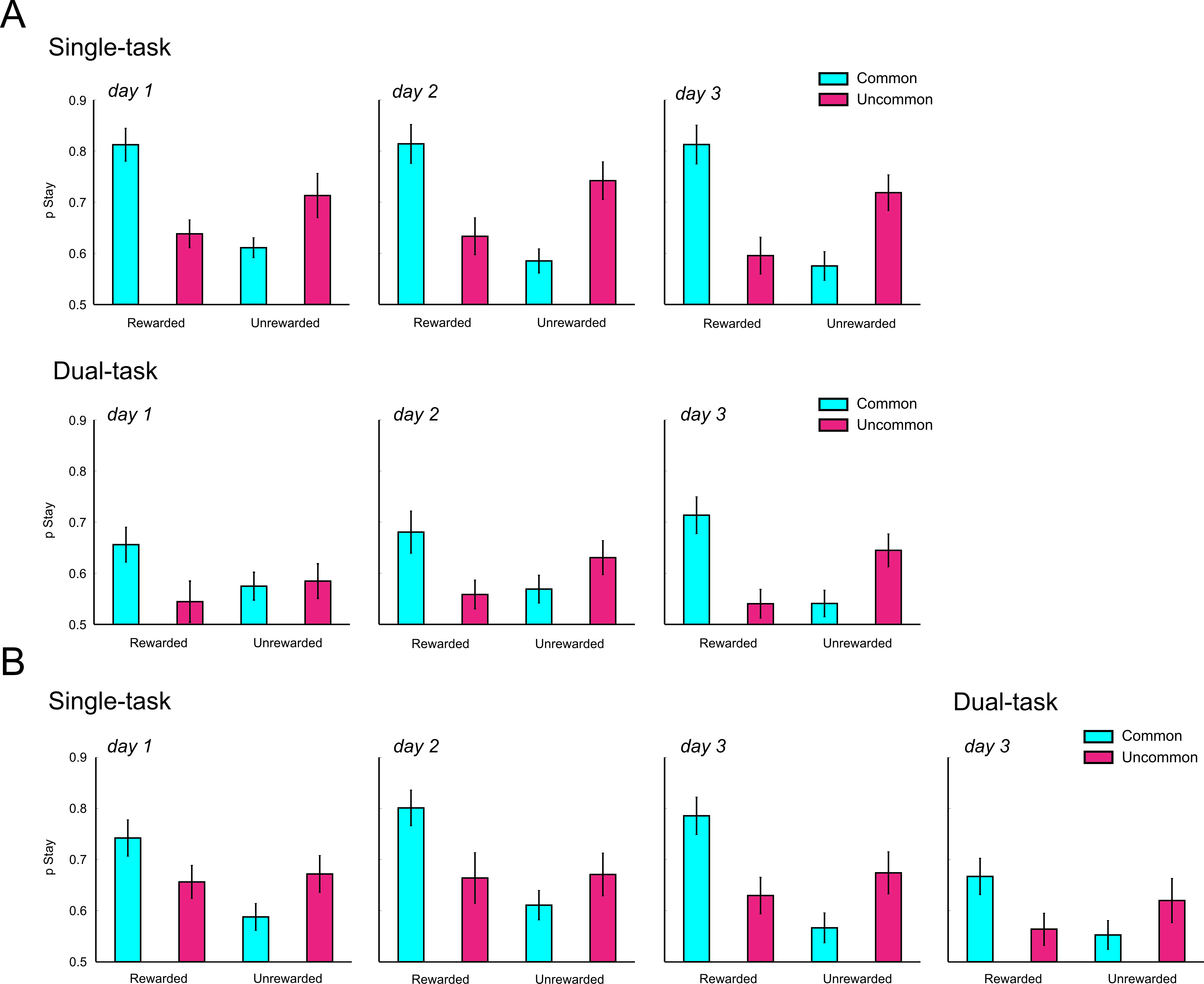

Supplement: S2 Fig — Bar plots show the average probability with which subjects chose to repeat their first-stage action on the subsequent trial as a function of the transition (common vs. uncommon) and outcome (rewarded vs. unrewarded) on the previous trial. Blue bars correspond to common transitions and red bars correspond to uncommon transitions. Vertical lines represent SEM. (A) Data from the ‘high load group’. The upper panel corresponds to the single-task condition and the lower panel to the dual-task condition. Choice is plotted separately for all 3 days. (B) Data from the ‘low load group’. Behavior is plotted across all 3 days for the single-task condition, and for day 3 alone in the dual-task condition. (TIF) [file pcbi.1004463.s002.tif]

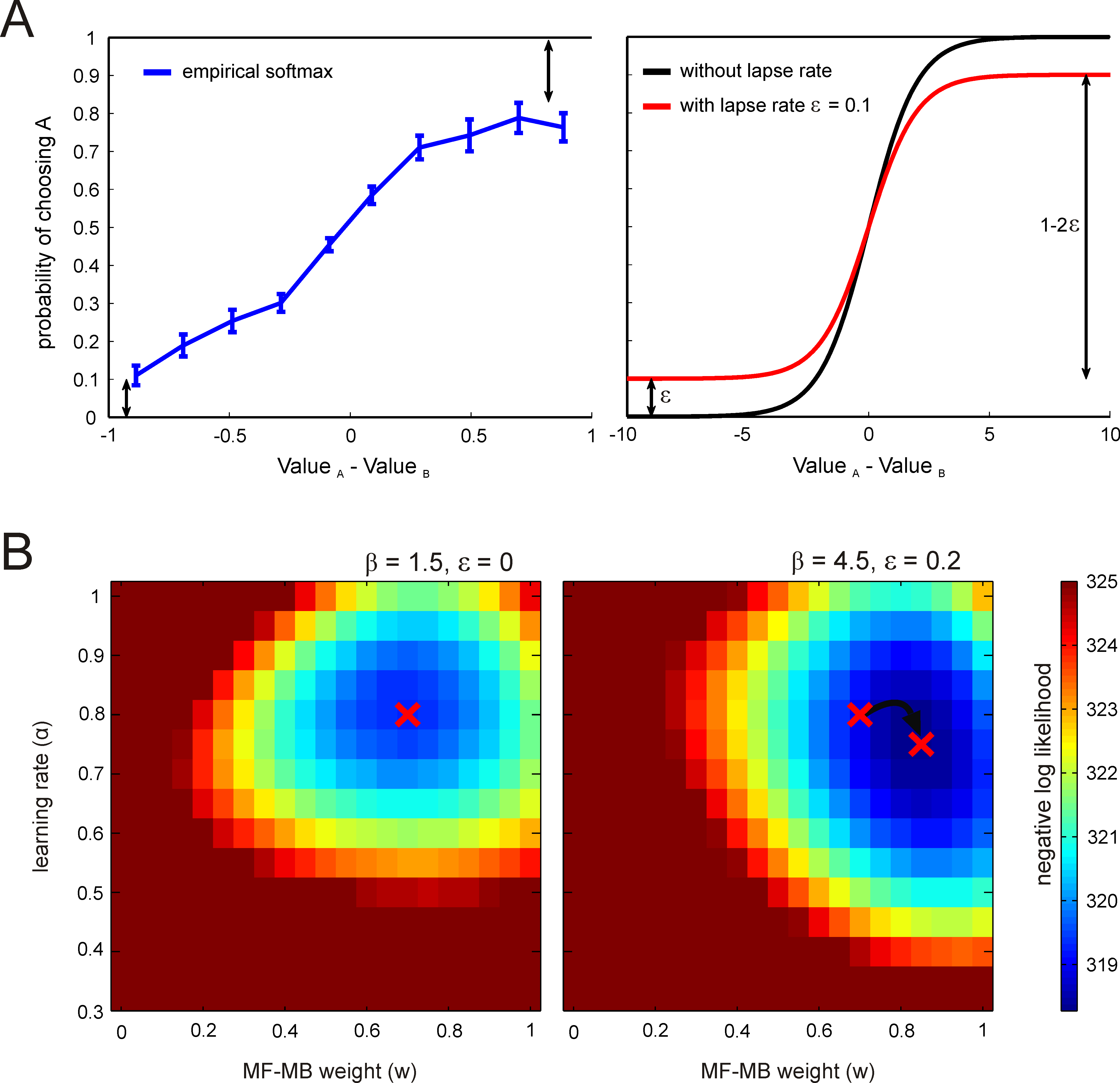

Supplement: S3 Fig — (A) The left-hand side shows an empirical softmax function generated using data from the ‘high load group’ on day 1 and the single-task condition. For each subject, we grouped the values generated from the winning hybrid model (see S1 Table) into 10 bins, and calculated the mean probability with which the best action was chosen in each bin, including both first and second-stage choices. The plot is averaged over all 22 subjects in the ‘high load group’. Vertical bars represent SEM. The right-hand side shows a simulated softmax function with an inverse temperature (β) of 1, with and without including a lapse rate (ε) set to 0.1. The lapse rate compresses the boundaries of the softmax such that the probability of choosing a given action is forced to lie between the range of 1-2ε. (B) Here we show slices through the likelihood surface of a single subject when the lapse rate (ε) is set to 0 (left-hand side), or fit as a free parameter (right-hand side), respectively. The red crosses represent the peak of the likelihood surface. On the right-hand side, the black arrow represents the shift in the peak of the surface (and the equivalent shift in the best-fitting values of our model parameters) when ε is fit as a free parameter compared to when it is fixed at 0. (TIF) [file pcbi.1004463.s003.tif]
