# Supplementary material for: Model-Based Reasoning in Humans Becomes Automatic with Training
Source: PLoS Comput Biol. 2015 Sep 17;11(9):e1004463. doi: 10.1371/journal.pcbi.1004463 (PMC4588166; doi:10.1371/journal.pcbi.1004463)
Supplement: S1 Table — Results of a Bayesian model comparison that accounted for differences in model complexity. The hybrid model, which incorporated influences from both model-free and model-based control, fit subject data better than pure model-free and model-based RL algorithms across both trial types (single-task versus dual-task) and both groups (‘high load group’ day 1, ‘low load group’ day 3). Bold-face denotes the winning model (lowest iBIC score) for each condition. α = learning rate; β = softmax inverse temperature; ε = lapse rate; w = model-free/model-based weight. The eligibility trace, λ (not shown), was set to 1 in all cases. w was set to 0 and 1 for pure model-free and pure model-based RL respectively. (DOCX) [file pcbi.1004463.s004.docx]

| *Models* | *iBIC single-task (x 10^4^)* | | *iBIC dual-task (x 10^3^)* | | *No. Parameters* |
| --- | --- | --- | --- | --- | --- |
|  |  |  |  |  |  |
|  | *High load group (day 1)* | *Low load group (day3)* | *High load group (day 1)* | *Low load group (day 3)* |  |
| α β ε (model-free) | 1.3831 | 1.4605 | 7.2260 | 7.8552 | 3 |
| α β ε (model-based) | 1.3688 | 1.4589 | 7.3056 | 7.8471 | 3 |
| α β ε w (hybrid) | **1.3491** | **1.4195** | **7.1863** | **7.8143** | 4 |

**Table S1: Bayesian model comparison: single days**. Results of a Bayesian model comparison that accounted for differences in model complexity. The hybrid model, which incorporated influences from both model-free and model-based control, fit subject data better than pure model-free and model-based RL algorithms across both trial types (single-task versus dual-task) and both groups (‘high load group’ day 1, ‘low load group’ day 3). Bold-face denotes the winning model (lowest iBIC score) for each condition. α = learning rate; β = softmax inverse temperature; ε = lapse rate; w = model-free/model-based weight. The eligibility trace, λ (not shown), was set to 1 in all cases. w was set to 0 and 1 for pure model-free and pure model-based RL respectively.
