# Supplementary material for: Model-Based Reasoning in Humans Becomes Automatic with Training
Source: PLoS Comput Biol. 2015 Sep 17;11(9):e1004463. doi: 10.1371/journal.pcbi.1004463 (PMC4588166; doi:10.1371/journal.pcbi.1004463)
Supplement: S2 Table — Results of a Bayesian model comparison that accounts for differences in model complexity. More complex model variants include those that have separate parameters for first and second stage choices, an eligibility trace, and a parameter for capturing shifts in model-free versus model-based control across days (σ). In simpler models, RL parameters were fixed between first and second stage choices, the eligibility trace was fixed at 1, and σ was set to 0. Bold-face denotes the winning model (lowest iBIC score) for each condition. Parameters followed by a superscript of 1 or 2 correspond to first-stage or second-stage choices respectively. α = learning rate; β = softmax inverse temperature; ε = lapse rate; w = model-free/model-based weight; λ = eligibility trace; σ = slope governing a shift in model-free/model-based weight (w) across days. (DOCX) [file pcbi.1004463.s005.docx]

| *Models* | *iBIC single-task (x 10^4^)* | | *iBIC dual-task*  *(x 10^4^)* | *No. Parameters* |
| --- | --- | --- | --- | --- |
|  | *High load group* | *Low load group* |  |  |
| α β ε w | 3.9598 | 4.2879 | 2.1602 | 4 |
| α β ε w λ | 3.9544 | 4.2880 | 2.1598 | 5 |
| α β ε w σ | 3.9703 | 4.2917 | 2.1598 | 5 |
| α β ε w λ σ | 3.9563 | 4.2912 | 2.1608 | 6 |
| α^1^ α^2^ β ε w | 3.9592 | 4.2877 | 2.1498 | 5 |
| α^1^ α^2^ β ε w λ | 3.9494 | 4.2887 | 2.1507 | 6 |
| α^1^ α^2^ β ε w σ | 3.9612 | 4.2907 | 2.1490 | 6 |
| α^1^ α^2^ β ε w λ σ | 3.9459 | 4.2906 | 2.1494 | 7 |
| α β^1^ β^2^ ε w | 3.9153 | 4.2556 | 2.1494 | 5 |
| α β^1^ β^2^ ε w λ | *3.9072* | **4.2494** | 2.1472 | 6 |
| α β^1^ β^2^ ε w σ | 3.9214 | 4.2612 | 2.1476 | 6 |
| α β^1^ β^2^ ε w λ σ | 3.9120 | 4.2541 | 2.1476 | 7 |
| α^1^ α^2^ β^1^ β^2^ ε w | 3.9134 | 4.2586 | 2.1449 | 6 |
| α^1^ α^2^ β^1^ β^2^ ε w σ | 3.9196 | 4.2632 | **2.1433** | 7 |
| α^1^ α^2^ β^1^ β^2^ ε w λ | **3.9055** | *4.2501* | *2.1442* | 7 |
| α^1^ α^2^ β^1^ β^2^ ε w λ σ | 3.9111 | 4.2569 | 2.1462 | 8 |

**Table S2: Bayesian model comparison: multiple days.** Results of a Bayesian model comparison that accounts for differences in model complexity. More complex model variants include those that have separate parameters for first and second stage choices, an eligibility trace, and a parameter for capturing shifts in model-free versus model-based control across days (σ). In simpler models, RL parameters were fixed between first and second stage choices, the eligibility trace was fixed at 1, and σ was set to 0. Bold-face denotes the winning model (lowest iBIC score) for each condition. Parameters followed by a superscript of 1 or 2 correspond to first-stage or second-stage choices respectively. α = learning rate; β = softmax inverse temperature; ε = lapse rate; w = model-free/model-based weight; λ = eligibility trace; σ = slope governing a shift in model-free/model-based weight (w) across days.
