# Supplementary material for: Model-Based Reasoning in Humans Becomes Automatic with Training
Source: PLoS Comput Biol. 2015 Sep 17;11(9):e1004463. doi: 10.1371/journal.pcbi.1004463 (PMC4588166; doi:10.1371/journal.pcbi.1004463)
Supplement: S3 Table — Best-fitting parameter estimates shown separately for each group and condition (single-task versus dual-task), using data concatenated across all 3 days of training. Values represent mean parameter fits across all subjects. * represents fixed parameter values. Parameters followed by a superscript of 1 or 2 correspond to first-stage or second-stage choices respectively. In simpler models, λ was fixed at 1 and σ was set to 0. α = learning rate; β = softmax inverse temperature; ε = lapse rate; w = model-free/model-based weight; λ = eligibility trace; σ = slope governing a shift in model-free/model-based weight (w) across days. (DOCX) [file pcbi.1004463.s006.docx]

| *Condition* | *α^1^* | *α^2^* | *β^1^* | *β^2^* | *ε* | *w* | *λ* | *σ* |
| --- | --- | --- | --- | --- | --- | --- | --- | --- |
|  |  |  |  |  |  |  |  |  |
| *High load group:*  *single-task* | 0.442 | 0.405 | 6.682 | 2.734 | 4.97 x10^-5^ | 0.723 | 0.546 | 0^*^ |
| *High load group:*  *dual task* | 0.097 | 0.355 | 3.981 | 2.250 | 0.021 | 0.899 | 1^*^ | -0.306 |
| *Low load group:*  *single-task* | 0.510 | | 4.752 | 2.560 | 6.66 x 10^-5^ | 0.566 | 0.628 | 0^*^ |

**Table S3: Inferred group-level parameters.** Best-fitting parameter estimates shown separately for each group and condition (single-task versus dual-task), using data concatenated across all 3 days of training. Values represent mean parameter fits across all subjects. * represents fixed parameter values. Parameters followed by a superscript of 1 or 2 correspond to first-stage or second-stage choices respectively. In simpler models, λ was fixed at 1 and σ was set to 0. α = learning rate; β = softmax inverse temperature; ε = lapse rate; w = model-free/model-based weight; λ = eligibility trace; σ = slope governing a shift in model-free/model-based weight (w) across days.
