# Supplementary material for: Model-Based Reasoning in Humans Becomes Automatic with Training
Source: PLoS Comput Biol. 2015 Sep 17;11(9):e1004463. doi: 10.1371/journal.pcbi.1004463 (PMC4588166; doi:10.1371/journal.pcbi.1004463)
Supplement: S4 Table — Table shows the group-level output of a logistic regression on first-stage switch-stay behavior, separately for single-task (‘high load group’ and ‘low load group’) and dual-task trials, from data concatenated across all 3 training sessions. We note that ‘reward x day’ was orthogonalized with respect to reward, and in turn ‘reward x transition x day’ was orthogonalized with respect to ‘reward x transition’. These regressors thus account for variance unexplained by the simpler main effect or 2-way interaction respectively (see Materials & Methods). Bold-face denotes p < 0.05 uncorrected for multiple comparisons. rew = reward; trans = transition. (DOCX) [file pcbi.1004463.s007.docx]

| *Regressor* | *Single-task* | | | | | | *Dual-task* | | |
| --- | --- | --- | --- | --- | --- | --- | --- | --- | --- |
|  | *High load group* | | | *Low load group* | | |  |  |  |
|  | *Estimate* | *SE* | *p-value* | *Estimate* | *SE* | *p-value* | *Estimate* | *SE* | *p-value* |
| intercept | -1.123 | 0.199 | **< 0.001** | -0.828 | 0.167 | **< 0.001** | -0.397 | 0.163 | **0.0235** |
| rew | -0.179 | 0.033 | **< 0.001** | -0.259 | 0.052 | **0.0001** | -0.058 | 0.030 | 0.0691 |
| trans | -0.139 | 0.084 | 0.1136 | 0.062 | 0.081 | 0.4510 | -0.084 | 0.073 | 0.2622 |
| day | 0.056 | 0.066 | 0.406 | -0.055 | 0.054 | 0.3219 | -0.057 | 0.055 | 0.3124 |
| rew * trans | -0.540 | 0.100 | **< 0.001** | -0.300 | 0.102 | **0.0081** | -0.278 | 0.052 | **< 0.001** |
| rew * day | 0.018 | 0.035 | 0.6105 | -0.085 | 0.048 | 0.0911 | -0.024 | 0.036 | 0.5164 |
| trans * day | 0.006 | 0.035 | 0.876 | -0.062 | 0.050 | 0.2234 | -0.002 | 0.036 | 0.9676 |
| rew * trans * day | -0.019 | 0.037 | 0.606 | -0.023 | 0.051 | 0.6599 | -0.110 | 0.052 | **0.0480** |

**Table S4: Results of a logistic regression across days.** Table shows the group-level output of a logistic regression on first-stage switch-stay behavior, separately for single-task (‘high load group’ and ‘low load group’) and dual-task trials, from data concatenated across all 3 training sessions. We note that ‘reward x day’ was orthogonalized with respect to reward, and in turn ‘reward x transition x day’ was orthogonalized with respect to ‘reward x transition’. These regressors thus account for variance unexplained by the simpler main effect or 2-way interaction respectively (see Materials & Methods). Bold-face denotes p < 0.05 uncorrected for multiple comparisons. *rew = reward*; *trans = transition*.
