# Supplementary material for: Model-Based Reasoning in Humans Becomes Automatic with Training
Source: PLoS Comput Biol. 2015 Sep 17;11(9):e1004463. doi: 10.1371/journal.pcbi.1004463 (PMC4588166; doi:10.1371/journal.pcbi.1004463)
Supplement: S1 Text — (DOCX) [file pcbi.1004463.s008.docx]

**Supporting Information**

Switch-stay choice strategy: 1-back regression

As standard in studies utilizing the two-step task [[1-8](#_ENREF_1)], we examined pairs of successive choices in isolation. Specifically, one can estimate the contribution of model-based versus model-free reasoning by means of a two-factor analysis of the effect of the previous trial’s reward and transition type respectively (common vs uncommon) on the first-stage choice (switch versus stay) in the current trial [[1](#_ENREF_1)]. Here, a model-free strategy predicts a player should repeat first-stage choices that led to rewards at the second-stage, regardless of the transition type (common or uncommon) between states (a main effect of reward on the probability of repeating the previous first-stage choice). By contrast, a model-based strategy predicts that a player should switch their first-stage choice if a reward is delivered after an uncommon transition, generating a higher probability of reaching a rewarding second-stage state (a cross-over reward x transition interaction in the probability of repeating the previous first-stage choice). Note these predictions arise from an assumption that subjects make choices based solely on events occurring in the immediately preceding trial, and thus provide only an approximate measure of the balance between model-free and model-based reasoning.

We performed a random-effects logistic regression, implemented in the Matlab software package (MathWorks), in which the dependent variable was the first-stage choices in the current trial (coded as 0 for stay, 1 for switch), and the explanatory variables included the reward and transition type on the previous trial (coded as 1 and -1), and their interaction. Blocks of the trials from the same day of training were concatenated, and trials where subjects failed to respond at either the first or second-stage were excluded from the analysis. When analyzing data across all days, we included a variable for the day of training, in addition to all possible interactions (see Table S4 for all variables). Here, our key interest lay in the 2-way interaction between reward and transition, and whether this interaction changes with training in the dual-task condition (a 3-way reward x transition x day interaction). Since these two regressors were highly correlated, we orthogonalized the latter with respect to the former, using a Gram-Schmidt process [[9](#_ENREF_9)]. Thus, any significant 3-way interaction represents a proportion of variance unaccounted for by a simple 2-way effect. One-sample t-tests were performed on all coefficients across subjects.

We first analyzed data from the ‘high load group’. Consistent with previous research [[1-3](#_ENREF_1),[6](#_ENREF_6)], we found that subjects’ first-stage choices on single-task trials were indicative of a mixture of both model-based and model-free reasoning on day 1 of training (Figure S2A). This choice strategy remained stable across (consecutive) days, with a logistic regression revealing both a main of effect of reward (all p < 0.01) and a cross-over reward x transition interaction (all p < 0.005) on all 3 days.

By contrast, both model-based and model-free choice was disrupted on day 1 of dual-task training (Figure S2A). Here, choice at the first-stage did not reveal a main effect of reward, and although we identified a reward x transition interaction (p < 0.05), it was not characterized by a full cross-over as predicted under a model-based strategy. Importantly however, subjects’ behavior in the dual-task condition shifted across days. We identified a cross-over reward x transition interaction (p < 0.003), but no main effect of reward, on days 2 and 3 of dual-task performance. Thus, on these days, subjects’ choices were consistent with the emergence of model-based control despite subjects performing the task under heavy cognitive load. When concatenating data across all 3 days, we identified a significant 3-way reward x transition x day interaction for dual-task trials (p < 0.05), but not single-task trials (p > 0.05), consistent with the proposal that training altered subjects’ performance under high but not low cognitive load (see Table S4).

When considering data from the ‘low load group’ we again observed a choice pattern indicative of both model-free and model-based reasoning across all 3 days of single-task training (main effect of reward, all p < 0.05; reward x transition interaction, all p < 0.05), but found no evidence for a change in behavior across days (reward x transition x day, p = 0.40) (Figure S2B). By contrast, dual-task performance on day 3 exhibited a cross-over reward x transition interaction (p < 0.001) but no main effect of reward (p = 0.59). These analyses suggest that task training renders model-based reasoning resistant to load, irrespective of whether training occurs in the presence or absence of load.

Adding lapse rates to softmax action selection

Greedily selecting the action currently believed best can lead to neglect of potentially better actions. Thus, some exploration in action selection is beneficial from a normative standpoint. Further, since subjects don't always behave deterministically in practice (whether for normative or other reasons), we need to account for noise in their choices. A common formulation of choice noise is to transform a value difference to a choice probability through an S-shaped curve (e.g., a logistic or "softmax" curve), such that the probability of selecting an action approaches 1 as its relative value increases.

However, softmax assumes all choice noise is value-dependent, with choices becoming asymptotically deterministic as the value difference between options increases. This assumption is often false in real behavior where value-independent sources of noise could include moments of inattention or distraction, pressing an unintended button, or valuation processes not captured by our model. One way to visualize this is by plotting the empirical link function between value difference and choice probability (Figure S3A; also cf. Figure 3 in [[10](#_ENREF_10)]). If probability does not asymptote to 1 (0) as value difference increases (decreases), then some choice noise is value-independent.

Thus, it can be useful to model this value-independent noise. The simplest form is ε-greedy selection [[11](#_ENREF_11)] where there is a 1-ε chance of selecting the highest-value action, and an ε chance of selecting a random action. This can be added trivially to standard softmax selection (see Figure S3A and ‘Action Selection’ in main text). Epsilon is sometimes called a "lapse rate" and is recently gaining traction in behavioral modeling [[10](#_ENREF_10),[12-14](#_ENREF_12)].

If value-independent noise is not accounted for, it will most likely interfere with estimation of model parameters. The intuition for this is that, in standard softmax, a single lapsed trial could be exceedingly unlikely (i.e., large negative log likelihood), even at the true parameters for the subject. A lapse rate softens the likelihood surface and permits a much smaller penalty for a few trials that don't fit with the rest. In Figure S3B, we plot two slices through the likelihood surface of an example subject’s choices for the winning hybrid model, when ***ε*** is set to 0 (left-hand slice, equivalent to standard softmax without lapse rate), or fit as a free parameter (right-hand slice). The crosses show the peak of the likelihood surface in each case. The black arrow shows a shift in this peak, and the corresponding best-fitting values of the learning rate (***α***), inverse temperature (***β***; not plotted) and model-free/model-based weight (***w)***, when ***ε*** is included in the model.

Supporting figure legends

**Figure S1: Model-free and model-based influences on choice: ‘low load group’.** We performed a logistic regression on data from the ‘low load group ’ on day 3 of training to estimate the relationship between choice on trial *t* and events occurring on trial *t_-1_* up to *t_-3_*. Here, regression coefficients can be interpreted as reflecting a model-free or model-based influence on choice, where larger coefficients indicate a stronger influence. In the single-task condition (blue bars), model-free and model-based coefficients were significantly different from 0 (up to 3 trials in the past), suggesting that subjects used a hybrid of both strategies. In the dual-task (high load) condition (orange bars), we observed a significant influence of a model-based system, that did not differ from the single-task condition, up to 3 trials in the past. In contrast, we found no significant influence of a model-free system. These results are consistent with data from the ‘high load group’ (see Figure 4). Vertical lines represent SEM. * denotes p =< 0.05, ‡ denotes p = 0.08.

**Figure S2: Switch-stay choice pairs.** Bar plots show the average probability with which subjects chose to repeat their first-stage action on the subsequent trial as a function of the transition (common vs. uncommon) and outcome (rewarded vs. unrewarded) on the previous trial. Blue bars correspond to common transitions and red bars correspond to uncommon transitions. Vertical lines represent SEM. (**A**) Data from the ‘high load group’. The upper panel corresponds to the single-task condition and the lower panel to the dual-task condition. Choice is plotted separately for all 3 days. (**B**) Data from the ‘low load group’. Behavior is plotted across all 3 days for the single-task condition, and for day 3 alone in the dual-task condition.

**Figure S3. The effect of utilizing a softmax lapse rate. (A)** The left-hand side shows an empirical softmax function generated using data from the ‘high load group’ on day 1 and the single-task condition. For each subject, we grouped the values generated from the winning hybrid model (see Table S1) into 10 bins, and calculated the mean probability with which the best action was chosen in each bin, including both first and second-stage choices. The plot is averaged over all 22 subjects in the ‘high load group’. Vertical bars represent SEM. The right-hand side shows a simulated softmax function with an inverse temperature (***β***) of 1, with and without including a lapse rate (***ε***) set to 0.1. The lapse rate compresses the boundaries of the softmax such that the probability of choosing a given action is forced to lie between the range of 1-2*ε*. **(B)** Here we show slices through the likelihood surface of a single subject when the lapse rate (***ε***) is set to 0 (left-hand side), or fit as a free parameter (right-hand side), respectively. The red crosses represent the peak of the likelihood surface. On the right-hand side, the black arrow represents the shift in the peak of the surface (and the equivalent shift in the best-fitting values of our model parameters) when ***ε*** is fit as a free parameter compared to when it is fixed at 0.

Supporting table legends

**Table S1: Bayesian model comparison: single days.** Results of a Bayesian model comparison that accounted for differences in model complexity. The hybrid model, which incorporated influences from both model-free and model-based control, fit subject data better than pure model-free and model-based RL algorithms across both trial types (single-task versus dual-task) and both groups (‘high load group’ day 1, ‘low load group’ day 3). Bold-face denotes the winning model (lowest iBIC score) for each condition. α = learning rate; β = softmax inverse temperature; ε = lapse rate; w = model-free/model-based weight. The eligibility trace, λ (not shown), was set to 1 in all cases. **w** was set to 0 and 1 for pure model-free and pure model-based RL respectively.

**Table S2: Bayesian model comparison: multiple days.** Results of a Bayesian model comparison that accounts for differences in model complexity. More complex model variants include those that have separate parameters for first and second stage choices, an eligibility trace, and a parameter for capturing shifts in model-free versus model-based control across days (σ). In simpler models, RL parameters were fixed between first and second stage choices, the eligibility trace was fixed at 1, and σ was set to 0. Bold-face denotes the winning model (lowest iBIC score) for each condition. Parameters followed by a superscript of 1 or 2 correspond to first-stage or second-stage choices respectively. α = learning rate; β = softmax inverse temperature; ε = lapse rate; w = model-free/model-based weight; λ = eligibility trace; σ = slope governing a shift in model-free/model-based weight (w) across days.

**Table S3: Inferred group-level parameters.** Best-fitting parameter estimates shown separately for each group and condition (single-task versus dual-task), using data concatenated across all 3 days of training. Values represent mean parameter fits across all subjects. * represents fixed parameter values. Parameters followed by a superscript of 1 or 2 correspond to first-stage or second-stage choices respectively. In simpler models, λ was fixed at 1 and σ was set to 0. α = learning rate; β = softmax inverse temperature; ε = lapse rate; w = model-free/model-based weight; λ = eligibility trace; σ = slope governing a shift in model-free/model-based weight (w) across days.

**Table S4: Results of a logistic regression across days.** Table shows the group-level output of a logistic regression on first-stage switch-stay behavior, separately for single-task (‘high load group’ and ‘low load group’) and dual-task trials, from data concatenated across all 3 training sessions. We note that ‘reward x day’ was orthogonalized with respect to reward, and in turn ‘reward x transition x day’ was orthogonalized with respect to ‘reward x transition’. These regressors thus account for variance unexplained by the simpler main effect or 2-way interaction respectively (see Materials & Methods). Bold-face denotes p < 0.05 uncorrected for multiple comparisons. *rew = reward*; *trans = transition*.

**References**

1. Daw ND, Gershman SJ, Seymour B, Dayan P, Dolan RJ (2011) Model-based influences on humans' choices and striatal prediction errors. Neuron 69: 1204-1215.

2. Wunderlich K, Smittenaar P, Dolan RJ (2012) Dopamine enhances model-based over model-free choice behavior. Neuron 75: 418-424.

3. Otto AR, Gershman SJ, Markman AB, Daw ND (2013) The curse of planning: dissecting multiple reinforcement-learning systems by taxing the central executive. Psychol Sci 24: 751-761.

4. Otto AR, Raio CM, Chiang A, Phelps EA, Daw ND (2013) Working-memory capacity protects model-based learning from stress. Proc Natl Acad Sci U S A 110: 20941-20946.

5. Skatova A, Chan PA, Daw ND (2013) Extraversion differentiates between model-based and model-free strategies in a reinforcement learning task. Front Hum Neurosci 7: 525.

6. Smittenaar P, FitzGerald TH, Romei V, Wright ND, Dolan RJ (2013) Disruption of dorsolateral prefrontal cortex decreases model-based in favor of model-free control in humans. Neuron 80: 914-919.

7. Smittenaar P, Prichard G, FitzGerald TH, Diedrichsen J, Dolan RJ (2014) Transcranial direct current stimulation of right dorsolateral prefrontal cortex does not affect model-based or model-free reinforcement learning in humans. PLoS One 9: e86850.

8. Voon V, Derbyshire K, Ruck C, Irvine MA, Worbe Y, et al. (2014) Disorders of compulsivity: a common bias towards learning habits. Mol Psychiatry.

9. Bjorck A (1994) Numerics of Gram-Schmidt Orthogonalization. Linear Algebra and Its Applications 198: 297-316.

10. Shteingart H, Neiman T, Loewenstein Y (2013) The role of first impression in operant learning. J Exp Psychol Gen 142: 476-488.

11. Sutton RSB, A. G. (1998) Reinforcement Learning: An Introduction: MIT Press, Cambridge, Massachusetts.

12. Talmi D, Dayan P, Kiebel SJ, Frith CD, Dolan RJ (2009) How Humans Integrate the Prospects of Pain and Reward during Choice. Journal of Neuroscience 29: 14617-14626.

13. Crockett MJ, Kurth-Nelson Z, Siegel JZ, Dayan P, Dolan RJ (2014) Harm to others outweighs harm to self in moral decision making. Proc Natl Acad Sci U S A 111: 17320-17325.

14. Guitart-Masip M, Economides M, Huys QJ, Frank MJ, Chowdhury R, et al. (2014) Differential, but not opponent, effects of L -DOPA and citalopram on action learning with reward and punishment. Psychopharmacology (Berl) 231: 955-966.
